# The convergence of Chinese county government health expenditures: capitation and contribution

**DOI:** 10.1186/s12913-016-1635-8

**Published:** 2016-08-19

**Authors:** Guoying Zhang, Luwen Zhang, Shaolong Wu, Xiaoqiong Xia, Liming Lu

**Affiliations:** 1 School of Public Administration, South China Normal University, Guangzhou, China; 2 School of Public Health, Sun Yat-sen University, Guangzhou, China; 3 Sun Yat-sen Global Health Institute, Sun Yat-sen University, Guangzhou, China; 4 Sun Yat-sen Center for Migrant Health Policy, Sun Yat-sen University, Guangzhou, China; 5 Department of Medical Administration, the 174th Hospital of the PLA, Chenggong Hospital of Xiamen University, Xiamen, China; 6 Key Unit of Methodology in Clinical Research, Guangdong Provincial Hospital of Chinese Medicine, Guangzhou, China

**Keywords:** Government health expenditure, Capitation, Convergence, County

## Abstract

**Background:**

The disparity between government health expenditures across regions is more severe in developing countries than it is in developed countries. The capitation subsidy method has been proven effective in developed countries in reducing this disparity, but it has not been tested in China, the world’s largest developing country.

**Methods:**

The convergence method of neoclassical economics was adopted to test the convergence of China’s regional government health expenditure. Data were obtained from *Provinces, Prefectures and Counties Fiscal Statistical Yearbook (2003–2007)* edited by the Chinese Ministry of Finance, and published by the Chinese Finance & Economics Publishing House.

**Results:**

The existence of σ-convergence and long-term and short-term β-convergence indicated the effectiveness of the capitation subsidy method in the New Rural Cooperative Medical Scheme on narrowing county government health expenditure disparities. The supply-side variables contributed the most to the county government health expenditure convergence, and factors contributing to convergence of county government health expenditures per capita were different in three regions.

**Conclusion:**

The narrowing disparity between county government health expenditures across regions supports the effectiveness of the capitation subsidy method adopted by China’s New Rural Cooperative Scheme. However, subsidy policy still requires further improvement.

## Background

At the 2005 World Health Assembly, all member states committed to achieving the goal of universal health coverage [1]. As is known, the most effective and equitable way to achieve universal health coverage is through obligatory funding such as taxation or social health insurance [2]. However, this requires allocating the raised funds equitably across regions. Both developed and developing countries to some extent face regional government health expenditure (GHE) disparities [3–5]. Health expenditure budgets, if based on historical expenditures or by government negotiation, enlarge the existing inequity, capitation subsidy is more effective in eliminating the disparity [6].

Capitation subsidy is defined as “the contribution to a plan’s budget associated with a plan member for the service in question for a given period of time.” Previous studies on the association between capitation subsidies and regional GHE equity mainly focus on developed countries. The WHO has reviewed the current formulas on the allocation of public funds to health expenditures in developed countries as well as the experiences and lessons from the practices of low-and-middle income countries [7]. England and Sweden have been exploring how to use weighted capitation subsidy methods to achieve equitable resource allocation, and their research has offered guidance for other countries [8]. Capitation subsidy methods have proven effective in improving the equity of GHE in developed countries [6, 7], but how effective have they been in developing countries? What are the influencing factors if capitation subsidy methods are used in developing countries? Some research has been conducted in developing countries, but this study explores the effectiveness and primary influencing factors of capitation subsidies on regional GHE equity in China, the world’s largest developing country.

The existing literatures on capitation subsidy in China have focused on regional equity of GHE. Chou & Wang analyzed the health expenditures of 28 provinces in China from 1978 to 2004, and found cluster convergence among regions with similar conditions, but no single convergence among provinces [9]. Pay et.al studied the equity of provincial GHE from 1997 to 2009, and found long-term convergence of GHE among provinces [10]. However, none of these studies has analyzed the impact of the change of allocation methods on regional equity. Furthermore, the analysis units have restricted to the provincial level, so the massive disparities among counties^1^ and within provinces have been neglected.

From 1990s to the early 2000s, regional inequality of health expenditures in China was severe. For instance, GHE per capita of several poor counties was less than 5 Yuan (1 US$ = 8.3 Yuan in 2003), while in other rich counties, it was over 200 Yuan. The disparity was caused by financing and allocation methods of county GHE before 2003. The majority of the county GHE came from county fiscal revenue; part was also from subsidies from superior governments (central, provincial and prefectural levels). Therefore, the local economic development and tax sharing system contributed to the disparity of county GHE per capita.

In China, regional economic development is imbalanced and the disparities are obvious. For example, in 2003, annual GDP per capita in the developed Southeastern coastal areas reached 50,000 Yuan, however the figure for undeveloped Central and Western areas remained less than 1000 Yuan. These more developed counties normally have higher county fiscal revenues. Meanwhile, the tax sharing system in China is designed to collect more fiscal revenues for higher-level government, and leave less to the county governments [11]. The transfer payment system primarily consists of upward and downward transfers between county governments and their superior government. Though the upward transfer allows the superior government to draw more funds from rich counties than the poor ones, the subsidy or distribution given by the superior government to county governments does not promote the equalization of county fiscal revenues. Instead, it enlarges the regional disparities of county fiscal revenues [12].

The grants appropriated to public service sectors are budgeted based on past actual expenditures [13]. As to the county GHE allocated from county fiscal revenue, the annual budget increased proportionally on the basis of local health expenditures in the previous years. This allocation method accepts the reasonability of previous years’ GHE, but does not improve regional GHE equity.

For transfer payments, the GHE subsidies from the superior governments are commonly directed as earmarked grants. The county governments have to compete for the limited funds, and will ultimately receive the appropriations through negotiations with the superior governments [14]. Because the developed regions have more political influence, the earmarked grants, except for those specifically assigned to the poverty-stricken counties, are usually appropriated to the developed regions, rather than to the developing regions. In other words, this allocation method may have been contributing to the county GHE disparity.

In addition to the massive regional GHE disparities, government investment in the health sector is also insufficient. The proportion of general GHE in China has dropped from 38.7 % in 1986 to 15.7 % in 2002. Meanwhile, the proportion of out-of-pocket health expenditures has increased significantly, from 26.4 % in 1986 to 57.7 % in 2002. To cope with the challenges, the Chinese government began to increase health investment in 2003, marked by the establishment of the New Cooperative Medical Scheme (NCMS) [15, 16].

NCMS, the health insurance scheme designed exclusively for Chinese rural residents, is administered by the county governments and financed jointly by governments and rural residents. As opposed to previous supply-side investments, the government switched to subsidizing demand. By doing so, the allocation method of the new fund was improved, and the NCMS fund was subsidized according to the number of enrollees. The NCMS financing policies released by the central government in 2003 stipulated that the governments must guarantee at least a 20 Yuan fiscal subsidy for each insured, and encourage all regions to invest more to satisfy the healthcare demands of rural residents. The undeveloped regions with lower fiscal revenues and more rural residents may undergo significant GHE increase. Yet, GHE in developed regions with fewer rural residents may experience a slower increase. Therefore, we would expect a narrowed GHE gap among county governments. In other words, the shift in allocation methods of the new fund from the traditional method to the capitation subsidy would significantly improve the equity of regional GHE in China.

### Framework

GHE per capita accounting for equity of health financing is affected by many factors. There are four approaches which influence how much governments spend on health [17]. The peer pressure approach recommends the amount of GHE for a government according to other governments with similar characteristics such as income or epidemiological profile. The political economy approach believes that health spending is influenced by the interplay of political and economic forces which determine budget priorities. The production function approach estimates changes in health spending based on health status after controlling other factors such as socioeconomic characteristics and demographics. The budget approach generates estimates of health spending through identifying health service demand and the price of health services.

Given a uniform tax rate for local governments, the disparities in healthcare services among local governments with different revenue levels would grow without national redistribution. All health systems need to allocate public funds to health plans in local government to fulfill their tasks in line with health policy. Five methods are usually used in budgeting public funds with government health expenditures included: (1) distribution originating from political patronage; (2) distribution based on historical precedent; (3) allocation by bids submitted by localities, or contingent on some measure of local performance; (4) expenditures localities actually spend; (5) allocation by mathematical formula [18]. The capitation subsidy method is the major approach of formula funding.

The capitation subsidy method may lead to more equitable allocation outcomes than the others. It consists of entities in reception of funds, health service benefit packages, number of people covered and risk adjustment [4]. By using the capitation subsidy method, the central government determines the scale of a grant received by health a plan in a local government according to the expected health service activities, as measured by the size and characteristics of the population in a locality [3, 4]. In actuality, the capitation subsidy method allocates a fixed budget to health plans in local governments based on population. This is more equitable than the other four methods.

In general, the capitation subsidy method allocates funds to health plans in local governments according to supply-side factors such as general revenues and grants, as well as demand-side factors such as demographics [5]. This is done in order to equalize the GHE in local governments and to reach health financing equity. According to the capitation subsidy method specifically, the historical GHE level, government general revenue, grants to county governments, transfers to superior the government, population density, sex ratio, and ratio of non-agricultural population were included in this research to test their influence on the delta of GHE per capita. We are not only concerned about whether or not the regional disparity decreases, but also the conditions and factors that narrowed the gap. The analysis of these factors has important implications to improving health financing policies.

## Methods

### Data

The Chinese government is divided into 5 levels— central, provincial, prefecture, county and township. In China’s healthcare system, the county government takes fundamental responsibility for providing healthcare services in rural areas. Due to large regional disparities, the county GHE is the most accurate representation of the equity of Chinese GHE, compared with the GHE of other government levels. In 2003, the capitation subsidy method was used to allocate fiscal directions to NCMS funds. This allocation method was also adopted by Urban Residents Basic Medical Insurance when it was established in 2007. Therefore, we used the panel data on county GHE from 2003 to 2007 to estimate the effect of capitation subsidies on narrowing county GHE disparities.

Between 2003 and 2007, there were 2861, 2862, 2862, 2860, and 2859 counties in China respectively. A total of 1941 counties were included in this study for the 5 consecutive years from 2003 to 2007, after excluding counties with missing values. The 9705 samples were subordinate to 31 provinces (including 22 provinces, 4 municipalities and 5 autonomous regions; Taiwan, Hong Kong and Macau were excluded). The fiscal revenues and GHE of the counties were extracted from Provinces, Prefectures and Counties Fiscal Statistical Yearbooks (quan guo di shi xian cai zheng tong ji zi liao) (2003–2007), edited by the Chinese Ministry of Finance, and published by the Chinese Finance& Economic Publishing House^2^; population figures were obtained from the China Population Statistical Yearbook (2004–2008); the geographic area of each county was obtained from the China Statistical Yearbook for Regional Economy 2001, and the consumer price index (CPI) was obtained from the China Statistical Yearbook (2004–2008). It should be noted that the data used in our study is household registration population, not resident population at the end of year. This is consistent with the NCMS capitation subsidy method in terms of household registration population. All yearbooks and data are publicly available in electronic and text format, and can be accessed from university libraries such as the Sun Yat-sen University Library.

### Variables and measurement

County GHE accounts for a large percentage of total government health expenditures, and is usually measured by GHE per capita. In this study, the control variables were categorized into demand-side and supply-side. The supply-side variables referred to the factors restricting county GHE, including the local government’s general revenue, grants from higher-level governments and transfers from county government to higher-level governments. Given the capitation subsidy method used in NCMS, the rural resident population was also an important variable. Since the contributions to NCMS funds from higher level governments could not be separated from the general subsidies, we used general subsidy to represent subsidies to NCMS funds in this study. We also included two demand-side variables: sex ratio and population density.

County GHE, general revenue, grants and transfers were divided by the county population to get the per capita values. They were then deflated by using the consumer price index (CPI), with 2003 as the base year. The rural resident population was conversely measured by the formula *1 - agricultural population/total population*, namely proportion of non-agricultural population, which reveals more straightforwardly the fast urbanization progress in China. Population density was calculated using population and geographic area, and sex ratio calculated as female population divided by the total population.

### Models

The disparity of regional GHE should be eliminated if the capitation subsidy method is effective. The σ-convergence model and β-convergence model are widely used to verify the changing tendency of regional economic disparity [19], and to test the hypothesis of the narrowing regional healthcare expenditure disparities [20]. Therefore, we tested the existence of σ-convergence and β-convergence of county GHE over time.

The concept of σ-convergence in neoclassical economics was initially defined as the tendency of dispersion of GDP per capita in a group of countries that declined over time [21]. In this study, σ-convergence indicated the declining tendency of dispersion of annual county GHE per capita from 2003 to 2007. It was measured by the coefficient of variation (CV) of county GHE per capita and the standard deviation(SD) of log county GHE per capita [20]. An F-test was used to verify the significance of convergence of CV and SD at the level of 5 % [22]. The models are showed as follows:

1 $$ \mathrm{C}{\mathrm{V}}_{\mathrm{t}+1}<C{\mathrm{V}}_{\mathrm{t}} $$

2 $$ \mathrm{S}{\mathrm{D}}_{\mathrm{t}+1}<S{\mathrm{D}}_{\mathrm{t}} $$

β-convergence was defined to demonstrate the faster economic growth of poor countries than rich countries. In this study, it represented the situation in which counties with lower initial GHE per capita in 2003 created faster growth rates than those with higher initial levels. The influencing factors on β-convergence are discussed as well. Baumol’s Conditional Convergence Model was adopted to test β-convergence (see formula 3) [23]. y_i,t_ was *i* county’s (i = 1,2,…,1941) logarithm of county GHE per capita in the year t (t = 2003,…,2007). Δy_i, t+k_ was the delta of the logarithm of county GHE per capita of county *i* during t + k period, and k was the time intervals (k = 1, 4). x_i, t_ referred to the vector of variables exerting influence on convergence including general revenue per capita, grant per capita, transfer per capita, ratio of non-agricultural population, sex ratio and population density. If x_i, t_ was excluded, the formula (3) referred to absolute β-convergence. If the value of β was negative, it was equal to the existence of absolute β-convergence and conditional β-convergence.

3 $$ \varDelta {\mathrm{y}}_{\mathrm{i},\mathrm{t}+\mathrm{k}}=\upalpha +\upbeta\ {\mathrm{y}}_{\mathrm{i},\mathrm{t}}+\upgamma\ {\mathrm{x}}_{\mathrm{i},\mathrm{t}}+{\upvarepsilon}_{\mathrm{i},\mathrm{t}} $$

## Results

### Descriptive analysis

A total of 1941 counties were included in the analysis (883 were excluded due to missing values). Mean and SD of each variable from 2003 to 07 were reported (see Table 1). The mean of GHE per capita, general revenue per capita, grants to county government per capita, and transfer to superior government per capita all increased from 2003 to 07: GHE per capita increased from 36.1952 Yuan in 2003 to 86.4012 Yuan in 2007, general revenue per capita from 339.3767 Yuan to 666.5741 Yuan, grants to county government per capita from 514.1106 Yuan to 1002.1680 Yuan, and transfer to superior government per capita from 69.6369 Yuan to 107.4491 Yuan. However, their SDs were large, exposing the disparities in health expenditures and fiscal revenues among counties. The ratio of non-agricultural population, population density and the sex ratio were also analyzed (see Table 1).

**Table 1:** Descriptive statistics of variables, 2003–07

Variable	2003	2004	2005	2006	2007
*n* = 1941	Mean (SD)	Mean (SD)	Mean (SD)	Mean (SD)	Mean (SD)
GHE per capita	36.1952	40.8757	45.4861	57.7739	86.4012
	(37.8421)	(80.6142)	(58.6031)	(58.0162)	(78.8955)
General revenue per capita	339.3767	408.3899	486.4796	572.7846	666.5741
	(440.2193)	(770.6048)	(815.8008)	(887.2564)	(1039.1240)
Grants to county government per capita	514.1106	624.0676	728.7735	862.1478	1002.1680
	(568.4750)	(923.0494)	(1251.0640)	(1020.3660)	(1240.1550)
Transfer to superior government per capita	69.6369	77.1887	90.9634	101.5136	107.4491
	(153.2010)	(213.9685)	(277.5415)	(365.3951)	(408.1884)
Ratio of non-agriculture population	0.2868	0.2989	0.3069	0.3106	0.3133
	(0.2620)	(0.2895)	(0.3054)	(0.2755)	(0.2746)
Population density	906.0498	935.8068	932.6604	940.5806	951.6450
	(2830.4420)	(3043.4190)	(2915.0320)	(2899.6320)	(2925.2370)
Sex ratio	0.4837	0.4839	0.4839	0.4842	0.4844
	(0.0108)	(0.0108)	(0.0107)	(0.0108)	(0.0109)

### σ-convergence

**Fig. 1:**
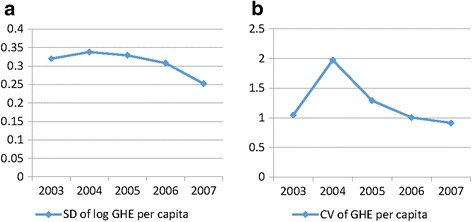
σ-convergence of county-level GHE per capita in China (2003–2007)

The changing tendency of SD and CV of county GHE per capita of 1941 counties from 2003 to 2007 is shown in Fig. 1. The SD of log county GHE per capita increased from 0.3197 to 0.3378 from 2003 to 2004, and then declined. CV showed a similar tendency. The convergence of county GHE per capita appeared beginning in 2005.

**Fig. 2:**
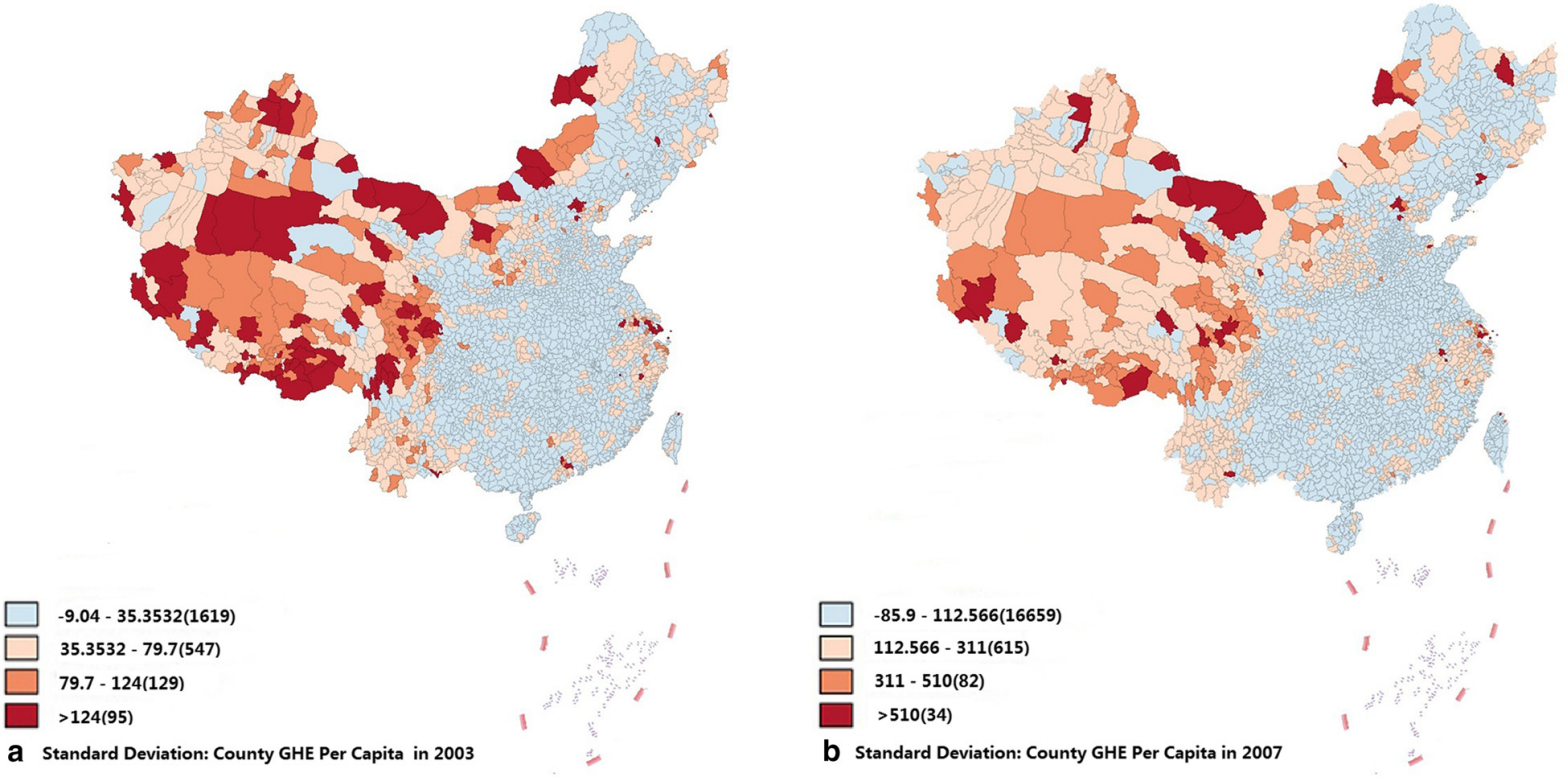
Distribution of SD of county-level GHE per capita in China in 2003 and 2007

The geographic distribution of the SD of Chinese county GHE per capita in 2003 and 2007 was analyzed with GIS mapping (see Fig. 2). The darker color, the larger SD. Fewer “dark-color counties” were shown in the 2007 map than in the 2003 map, indicating the shrinking disparity of county GHE.

An F-test was used to further test the convergence of SD and CV in county GHE. From 2005 onward, the SD of annual county GHE per capita was significantly less than that of the previous year, confirming the convergence (see Table 2). The test results of CV of county GHE per capita were similar to those of SD. To summarize, σ-convergence existed in Chinese county GHE from 2003 to 2007, indicating the success of NCMS the capitation subsidy method in narrowing regional disparities. During the initial period of NCMS implementation (2003–2004), the capitation subsidy method did not exert obvious effects, however it did show significant effects after 2005.

**Table 2:** σ-convergence and F-test

Year	SD of Log GHE	F-value	CV of GHE	F-value
2003	0.3197	—	1.0455	—
2004	0.3378	0.8956	1.9722	0.281
2005	0.3289*	1.0552	1.2884*	2.3432
2006	0.3083*	1.1376	1.0042*	1.6461
2007	0.2520*	1.4976	0.9131*	1.2094

Note: F-test for SD (Microsoft Excel 2007 software)_,_ =SD_t-1_^2^/SD_t_^2^, where t-1 (t = 2004, …, 2007) is the base year, and t is the year under test. The null hypothesis is SD_t-1_^2^ ≤ SD_t_^2^. The F-test for CV is the same. * is significance at 5 %. SD and CV refer to standard deviation and coefficient of variation, respectively. GHE is the abbreviation of government health expenditure

### β-convergence

**Fig. 3:**
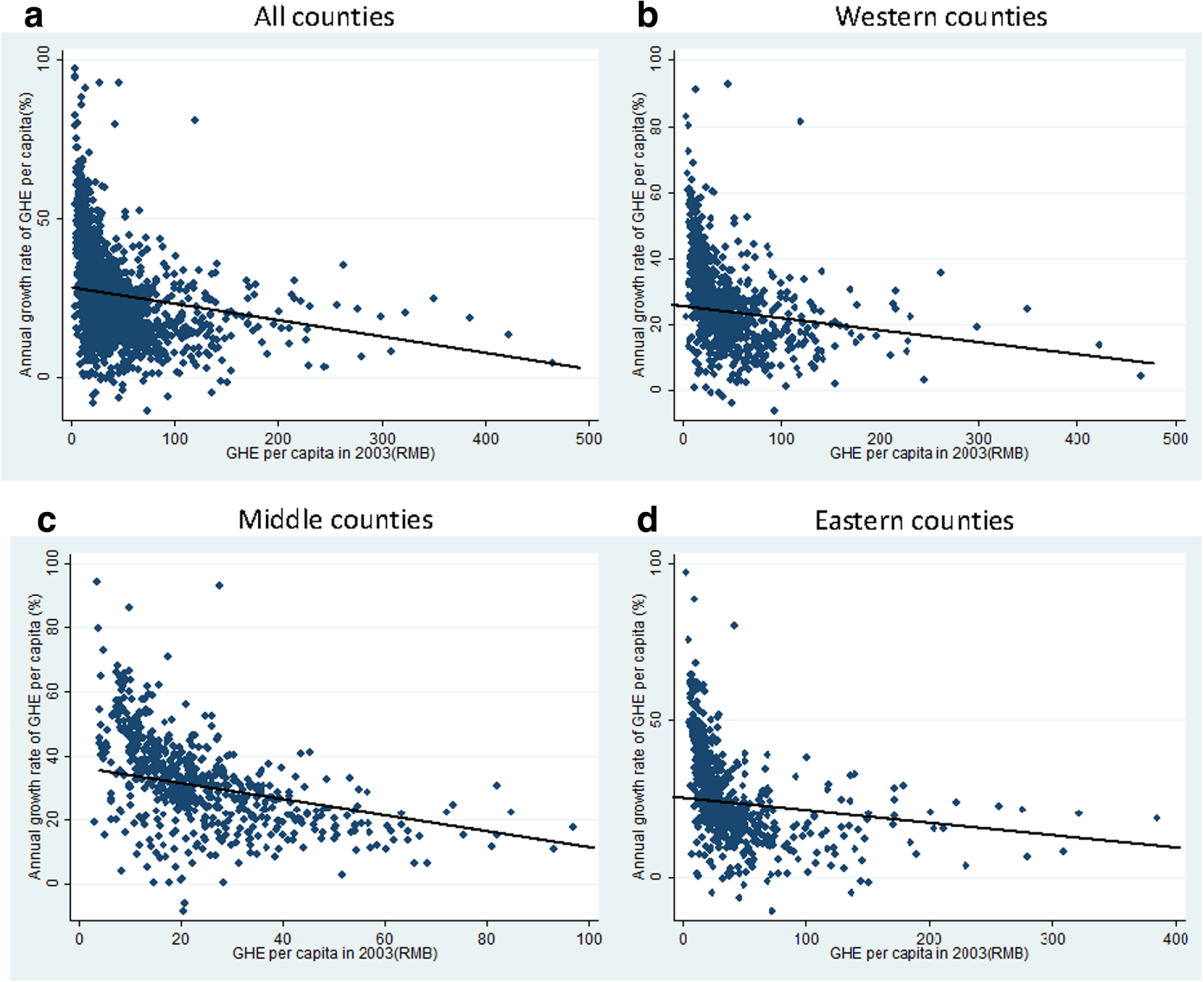
GHE per capita in 2003 and annual growth rate of GHE per capita from 2003 to 2007. Figure (**a**) presents all counties in China, and (**b**, **c**, **d**) presents the counties in the West, Central, and Eastern regions of China, respectively

In this study, 1941 counties were divided into 3 regions (East, Central and West) according to definitions given by the China National Bureau of Statistics. The scatter diagrams manifested the relationship of initial county GHE per capita in 2003, as well as the GHE per capita growth rate during the period from 2003 to 2007 (see Fig. 3). All four diagrams showed the negative correlation both in the 3 separate regions and nationwide. Counties with lower initial county GHE per capita had faster growth rates than those with higher initial levels. The preliminary observation of the scatter diagram suggested the existence of β-convergence.

According to time intervals (k = 1, 4) and convergence types (absolute β-convergence and conditional β-convergence), we established 4 statistical models to test β-convergence: (see Table 3) (1) short-term absolute β-convergence, (2) short-term conditional β-convergence, (3) long-term absolute β-convergence and (4) long-term conditional β-convergence. The coefficient of GHE per capita was significantly negative at the level of 0.1 % in all models, suggesting the existence of long-term and short-term β-convergence. The marginal effect of GHE per capita on regional disparity reduction was 9.88, 24.10, 35.50 and 54.50 % respectively from model (1) to model (4). The findings indicated that the counties with lower initial GHE levels not only had higher growth rates than those with higher initial level, but also contributed more to the reduction of regional GHE disparity.

**Table 3:** Regression results of β-convergence

Delta GHE	(1)	(2)	(3)	(4)
Log GHE	−0.0988^***^	−0.2410^***^	−0.3550^***^	−0.5450^***^
	(0.0057)	(0.0099)	(0.0103)	(0.0157)
Log general revenue		0.0324^***^		0.0789^***^
		(0.0072)		(0.0131)
Log grants to county government		0.2010^***^		0.2820^***^
		(0.0103)		(0.0170)
Log transfer to superior government		−0.0187^***^		−0.0190^***^
		(0.0034)		(0.0056)
Population density		0.000002^*^		0.000005^***^
		0.0000		0.0000
Sex ratio		−0.0038		0.0065
		(0.0061)		(0.0133)
Ratio of non-agriculture population		−0.0131		−0.1980^***^
		(0.0075)		(0.0146)
Constant	0.2540^***^	−0.1210^***^	0.9300^***^	0.3530^***^
	(0.0088)	(0.0235)	(0.0151)	(0.0405)
*N*	7764	7764	1941	1941
R-squared	0.0374	0.0868	0.3805	0.5158

Note: Each column represents a regression based on equation (3). Column (1) and (2) test the short-term β-convergence (k = 1), and Column (3) and (4) test the long-term β-convergence (k = 4). *, *** is significant at 5 %, 0.1 %, respectively. Standard errors are in parentheses. Model (1) and (3) are estimated by univariate Ordinary Least Square (OLS) method. Model (2) and (4) are estimated by multivariate General Least Square (GLS) and OLS method, respectively (Stata 11 software). GHE is the abbreviation of government health expenditure

The supply-side variables were analyzed first. In model (2) and (4), the coefficients of general budget revenues and of grants to county government were significantly positive at the level of 0.1 %, indicating that the two factors enlarged the county GHE per capita disparity. Grants to county government were commonly designed to promote regional equalization, but the results in our study showed a reverse effect. This finding alerted us that China’s fiscal subsidy policy needed improvement.

The coefficients of transfer to superior government and of ratio of non-agriculture population were significantly negative at the level of 0.1 % in the long-term, suggesting its effectiveness in reducing the disparity of county GHE per capita. Transfer to the superior government reduced county fiscal revenue disparities, and therefore reduced the county GHE per capita disparity. Ratio of non-agriculture population, representing the level of urbanization, also contributed to the reduction of the county GHE per capita disparity.

For the demand-side variables, population density increased county GHE per capita disparities at the level of 5 % in the short-term and, at the level of 0.1 % in the long-term, but with a weak marginal effect. The effect of sex ratio was not statistically significant. In general, during 2003–2007, the supply-side factors contributed more to county GHE per capita disparities than did the demand factors.

Below is the analysis of regional disparities of county GHE per capita in Eastern, Central and Western China (see Table 4). The coefficients of county GHE per capita in the 3 regions were all significantly negative at the 0.1 % level, indicating the existence of β-convergence among regions. The findings were consistent with the nation-level analysis.

**Table 4:** Regression results of β-convergence in three regions of China

Delta Per GHE	Western region				Central region				Eastern region			
	(1)	(2)	(3)	(4)	(1)	(2)	(3)	(4)	(1)	(2)	(3)	(4)
Log GHE	−0.0883^c^	−0.2370^c^	−0.3240^c^	−0.5170^c^	−0.1290^c^	−0.2620^c^	−0.4330^c^	−0.6220^c^	−0.0944^c^	−0.2570^c^	−0.3530^c^	−0.5730^c^
	(0.0086)	(0.0145)	(0.0154)	(0.0242)	(0.0146)	(0.0231)	(0.0260)	(0.0319)	(0.0091)	(0.0175)	(0.0175)	(0.0286)
Log general revenue		−0.0012		0.0251		0.0381		0.2170^c^		0.0949^c^		0.1630^c^
		(0.0097)		(0.0193)		(0.0204)		(0.0390)		(0.0137)		(0.0240)
Log grants to county government		0.2200^c^		0.2870^c^		0.2040^c^		0.2680^c^		0.1780^c^		0.2500^c^
		(0.0149)		(0.0252)		(0.0269)		(0.0449)		(0.0169)		(0.0284)
Log Transfer to superior government		−0.0139^c^		0.0010		0.0027		−0.0291		−0.0485^c^		−0.0673^c^
		(0.0042)		(0.0069)		(0.0106)		(0.0152)		(0.0070)		(0.0130)
Population density		0.000001		0.000001		0.000001		0.000003		0.000002^a^		0.000007^c^
		0.0000		0.0000		0.0000		0.0000		0.0000		0.0000
Sex ratio		−0.0098		0.0012		−0.0051		0.1800		0.0003		0.2080
		(0.0114)		(0.0131)		(0.0136)		(0.1475)		(0.0083)		(0.1121)
Ratio of non-agriculture population		−0.0021		−0.1780^c^		−0.0135		−0.2390^c^		−0.0307^a^		−0.1980^c^
		(0.0123)		(0.0261)		(0.0153)		(0.0360)		(0.0123)		(0.0207)
Constant	0.2410^c^	−0.1110^b^	0.8940^c^	0.4000^c^	0.2980^c^	−0.1370^a^	1.0350^c^	0.0182	0.2430^c^	−0.1460^c^	0.9100^c^	0.1230
	(0.0137)	(0.0349)	(0.0234)	(0.0602)	(0.0206)	(0.0632)	(0.0342)	(0.1859)	(0.0143)	(0.0376)	(0.0261)	(0.1345)
*N*	3156	3156	789	789	1988	1988	497	497	2620	2620	655	655
R-squared	0.0325	0.1017	0.3610	0.4950	0.0381	0.0707	0.3580	0.5098	0.0393	0.0988	0.3850	0.5450

Each column represents a regression based on equation (3) in three regions. Column (1) and (2) tests the short-term β-convergence (k = 1), and Column (3) and (4) tests the long-term β-convergence (k = 4) in three regions. ^a^, ^b^, ^c^ is significant at 5 %, 1 %, 0.1 %, respectively. Standard error is in parentheses. Model (1) and (3) are estimated by univariate Ordinary Least Square (OLS) method. Model (2) and (4) are estimated by multivariate General Least Square (GLS) and OLS method, respectively (Stata software 11). GHE is the abbreviation of government health expenditure

For the supply-side variables, general budget revenue did not significantly influence the intra-regional county GHE per capita disparity in the West, but it did enlarge it in the Central and Eastern regions. Grants to county government enlarged intra-regional county GHE per capita disparity in all regions, which again confirmed the necessity of improving China’s fiscal subsidy policy. Transfer to superior governments exerted a complicated effect. In the Western regions, it significantly reduced the county GHE per capita disparity in the short-term, but did not exert long-term influence. Transfer contributed significantly to the elimination of the county GHE per capita disparity in the East, but did not work in Central China.

The ratio of non-agricultural population showed significant long-term influence on county GHE per capita disparity in all three regions at the level of 0.1 %, but the short-term influence was only observed in the Eastern region. Among demand-side variables, population density raised county GHE per capita disparity only in the East, while sex ratio did not show significant influence in any of the three regions.

To summarize, β-convergence of county GHE per capita existed in all three regions of China. Each region was influenced by different influencing factors, and more factors contributed to county GHE per capita disparity in the Eastern region than in the Central and Western regions. We also used a mixed regression model, a random effects model, and a fixed effects model to test the significance of the results in the short-term models, as well as a robust regression to test the significance of the results in the long-term models. The results of explaining convergence of GHE per capita with these variables was robust, but the results of the robust check are not presented in the table due to space limitations.

## Discussion

Tendency analysis and an F-test showed fast σ-convergence of county GHE since 2005, indicating the effectiveness of the capitation subsidy method adopted by NCMS, and the success in narrowing the disparities of county GHE per capita. NCMS was piloted in 2003 in approximately 100 counties, and the government subsidized 20 Yuan for each insured. In 2007, NCMS were established in 2452 counties, serving 726 million (86.20 %) rural residents. The government subsidies then increased to 60 Yuan per capita, accounting for 60 % of GHE per capita. The increasing government subsidies and NCMS coverage not only increased the county GHE, but also promoted the equalization of county GHE per capita.

Previous studies have argued that the convergence was caused by the switch from subsidizing the supply-side to subsidizing the demand-side [16]. However, our study argues that the switch itself did not automatically lead to GHE per capita convergence. The capitation subsidy method is the real power that promotes the elimination of regional GHE disparity, regardless of which side the government subsidizes. For example, China’s government employee insurance system (GIS) subsidizes the demand-side via a fee-for-service method that leads to large regional disparities of GIS medical expense per capita. National Health Service (NHS) in the U.K. uses the capitation subsidy method to subsidize the supply-side, and regional disparities in GHE per capita are limited.

Both β-convergence and σ-convergence analysis showed similar tendencies of convergence of county GHE per capita in the short-term and the long-term. This indicates that the capitation subsidy method in NCMS has rapidly reduced disparities of county GHE per capita. Furthermore, it shows that counties with lower initial spending levels had faster growth rates in county GHE per capita than the counties with higher initial spending levels.

As an aspect of county fiscal investment in NCMS funds, the policies set out the lowest appropriation levels and encouraged the richer counties to invest more. The general revenue significantly enlarged the disparity of county GHE per capita, which indicates that the developed areas allocate more health expenditures to their residents. The results of our study support the widely-acknowledged viewpoint that health expenditures are both a national luxury and an individual necessity [24, 25].

The subsidies from higher-level governments only enlarged county GHE per capita disparities. This analysis coincides with previous studies, and indicates that China’s grant policies need immediate improvement [12]. As for the subsidies to NCMS funds, in 2003, the central government subsidized 10 Yuan per year for each insured in the less-developed Central and Western China (except municipal districts), and the local governments were required to subsidize no less than 10 Yuan. However, in the developed Eastern China (e.g., Guangdong Province), the central government did not offer any subsidies. However, subsidization based on geographic regions (East, Central, and West) may neglect intra-regional disparity, as it did not contribute to improving GHE equity among counties. The central government indirectly subsidizes the counties in the Central and Western regions based on provincial general revenues according to NCMS policy. This policy neglects the intra-provincial distinctions among counties, and therefore enlarges the disparity of county GHE per capita. The central government should directly subsidize NCMS funds based on county general fiscal revenue, which would promote the equalization of county GHE per capita.

The regions that are undeveloped or have limited fiscal revenue usually have more rural residents, so they can acquire more NCMS subsidies to achieve a faster increase of county GHE per capita. Urbanization contributes to the reduction of the county GHE per capita disparity, reflecting the long-term dispersed health policies towards rural residents in China. The ongoing large-scale urbanization and population migration will continue to facilitate the narrowing of the county GHE per capita disparity by decreasing the number of rural residents [26].

The variables on the supply-side have the most significant effect on reducing county GHE per capita disparities. This indicates that the NCMS subsidy policy first considers the government’s financial capacity, rather than the demands of the population’s health. The allocation formula for the capitation subsidy method should be improved according to both supply-side and demand-side factors in order to allocate NCMS funds more equitably. Nothing but equitable allocation of GHE across counties can lead to the realization of universal health coverage.

The long-term and short-term convergence of county GHE per capita exists on both the regional and national levels, and the capitation subsidy method also has improved intra-regional equity. In the Western regions, general revenues of most counties are comparatively low. Therefore, the county governments generally accomplish their policy standards, and few of them are willing to invest more to reduce the county GHE per capita disparity. In contrast, in the Central and Eastern regions, the richer county governments have the willingness and fiscal capacity to invest more. This causes larger county GHE per capita disparities. One factor may cause multiple effects in different regions. This implies the complexity of the implementation of the NCMS policy. To achieve equity in county GHE per capita, we need to explore the mechanisms of health policy implementation in various regions.

This paper has some limitations. First of all, the ultimate objective of the convergence of GHE per capita is to improve residents’ healthcare utilization and health outcomes. However, due to space limitations, we have focused on the effectiveness of capitation subsidy methods on the equity of GHE and its influencing factors. More efforts should be conducted to explore how the equalization of GHE per capita influences healthcare utilization and health outcomes. Secondly, although fund flows between county government, prefecture government, provincial government and central government (including transfers from county governments to superior governments and grants from superior governments to county governments) are also an important topic to clarify, the subsidy institutions and health programs in different provinces are complicated and diverse. This paper has clarified the transfer payment relationship by way of example within a limited space. Further research should discuss the roles and mechanism of health fund flows between different levels of government.

## Conclusion

Universal health coverage requires equitable distribution of GHE to each county. The capitation subsidy method has proven effective in improving the equity of regional GHE. China established the NCMS scheme in 2003 and since then has used the capitation subsidy method to distribute public funds to NCMS funds in counties. The analysis of county GHE data (2003–07) verified the existence of σ-convergence and short-term and long-term β-convergence at the national level. It has been shown that the capitation subsidy method used in NCMS has rapidly reduced county GHE per capita disparities, and has improved county equity.

Transfers to higher level governments and a decreasing agricultural population has reduced the county GHE per capita disparities. Meanwhile, general revenues and grants from higher level governments exert the opposite effect. China’s present capitation subsidy method merely considers supply-side factors, but neglects the effects of demand-side factors. The factors contributing to the convergence of county GHE per capita are different in the three regions, which indicate the existence of different implementation mechanisms for NCMS policy.

## Abbreviations

CPI: Consumer Price Index
GHE: Government Health Expenditures
GIS: Government Employee Insurance System
NCMS: New Cooperative Medical Scheme
NHS: National Health Service

## Footnotes

1 In this paper, county refers to county-level units including counties, autonomous counties, county-level cities, qi, autonomous qi, and districts.

2 Based on Local Fiscal Final Accounts (di fang cai zheng zong jue suan) (2003–2007), the data in Provinces, Prefectures and Counties Fiscal Statistical Yearbook (2003–2007) was collected, calculated, and aggregated. Furthermore, the data in Local Fiscal Final Accounts originated from the local government’s routine record of fiscal revenue and expenditure.

## References

[1] WHO. Sustainable health financing, universal coverage and social health insurance. Geneva: World Health Assembly Resolution; 2005.

[2] WHO. The world health report-health systems financing: the path to universal coverage. Geneva: World Health Organization; 2010. 10.2471/BLT.10.078741 PMC2878164 20539847

[3] Giannoni M, Hitiris T (2002). The regional impact of health care expenditure: the case of Italy. Appl Econ.

[4] Di Matteo L, Di Matteo R (1998). Evidence on the determinants of Canadian provincial government health expenditures: 1965–1991. J Health Econ.

[5] Pan J, Liu GG (2012). The determinants of Chinese provincial government health expenditures: evidence from 2002–2006 data. Health Econ.

[6] Rice N, Smith PC (2001). Capitation and risk adjustment in health care financing: an international progress report. Milbank Q.

[7] Smith PC. Formula funding of health services: learning from experience in some developed countries. Geneva: World Health Organisation; 2008.

[8] Diderichsen F, Varde E, Whitehead M (1997). Resource allocation to health authorities: the quest for an equitable formula in Britain and Sweden. BMJ.

[9] Chou WL, Wang Z (2009). Regional inequality in China’s health care expenditures. Health Econ.

[10] Pan J, Wang P, Qin X, Zhang S (2013). Disparity and Convergence: Chinese Provincial Government Health Expenditures. PLoS One.

[11] Wong CP (2000). The 1994 tax-sharing reform and public expenditure management in China.

[12] Heng Y (2008). Fiscal disparities and the equalization effects of fiscal transfers at the county level in China. Ann Econ Financ.

[13] Ma J (2006). Zero-based budgeting in China: experiences of Hubei province. J Public Budgeting Account Financ Manage.

[14] Liu M, Wang J, Tao R, Murphy R (2009). The political economy of earmarked transfers in a state-designated poor county in western China: central policies and local responses. China Q.

[15] Tang S, Meng Q, Chen L, Bekedam H, Evans T, Whitehead M (2008). Tackling the challenges to health equity in China. Lancet.

[16] Liu Y, Rao K, Hsiao WC (2003). Medical expenditure and rural impoverishment in China. J Health Popul Nutr.

[17] Savedoff W (2005). How much should countries spend on health?.

[18] Smith PC. Formula funding of public services. New York: Routledge; 2006.

[19] Barro RJ, Sala-i-Martin X. Economic growth and convergence across the United States. Cambridge: National Bureau of Economic Research; 1990.

[20] Hitiris T, Nixon J (2001). Convergence of health care expenditure in the EU countries. Appl Econ Lett.

[21] Dalgaard C, Vastrup J (2001). On the measurement of σ-convergence. Econ Lett.

[22] Nixon J. Convergence of health care spending and health outcomes in the EUropean Union, 1960–95. York: University of York, Centre for Health Economics; 2000.

[23] Baumol WJ. Productivity growth, convergence, and welfare: what the long-run data show. Am Econ Rev. 1986;76(5):1072–1085.

[24] Getzen TE (2000). Health care is an individual necessity and a national luxury: applying multilevel decision models to the analysis of health care expenditures. J Health Econ.

[25] Newhouse JP. Medical-care expenditure: a cross-national survey. J Hum Resour. 1977;12(1):115–125. 404354

[26] Gong P, Liang S, Carlton EJ, Jiang QW, Wu JY, Wang L, Remais JV (2012). Urbanisation and health in China. Lancet.

